# O-band DWDM data transmission with quantum dot mode-locked comb laser and semiconductor optical amplifier

**DOI:** 10.1038/s41598-026-46147-z

**Published:** 2026-04-18

**Authors:** Vasilii V. Belykh, Mikhail S. Buyalo, Janina Rautert, Sergey S. Mikhrin, Vladimir S. Mikhrin, Artem V. Zhabotinskii, Alexey R. Kovsh, Alexey E. Gubenko

**Affiliations:** 1 Innolume GmbH, 44263 Dortmund, Germany; 2 Alfalume Inc, Los Gatos, CA USA

**Keywords:** Optics and photonics, Physics

## Abstract

There is a long-standing prospect of using a single comb laser chip emitting multiple low-noise spectral modes for DWDM data transmission in the O-band. This goal is hindered by unacceptably high total power requirements for such a source to overcome losses in a PIC and to provide at least 1 mW per data channel in a fiber. We solve this problem by simultaneously re-amplifying more than 20 lines depleted after the PIC using a low-noise quantum dot (QD)-based SOA. In this way, we demonstrate O-band DWDM data transmission of a PAM4 signal with a total bit rate of up to 2.3 Tb/s in fiber with a single QD comb laser source. The other problem related to the application of semiconductor comb lasers is the insufficiently large interline separation to be compatible with the state-of-the-art DWDM PIC technologies. We present comb laser devices with and without a saturable absorber section, providing up to 23 lines with 100 GHz intermode separation, 10 lines with 138 GHz separation, 4 lines with 163 GHz separation, and 3 lines with 216 GHz separation, suitable for data transmission. We show that in the mode-locking regime, both the relative intensity noise (RIN) of the comb laser and the associated bit error rate (BER) are determined by the optical power of the laser mode.

## Introduction

In the last few years, the data transmission capacity has experienced explosive growth stimulated by the development of artificial intelligence and machine learning (AI/ML) technologies, which require massive clusters for parallel computation with millions of interconnects. The other tendency is the onset of optical fiber communications, which are starting to dominate over electrical interconnects. In former days, optical fibers were mostly used for connections spanning over several kilometres. Now they are massively used to connect servers within clusters over distances of a few tens of meters and in the near future, optical cables will be used for interconnects within a server over distances of about 1 m. The decrease in the required interconnect distances is accompanied by the dramatic increase of the transmission capacity and therefore by the number of required transceivers.

The growing demand for data transmission capacity requires an increase in data bandwidth within a single fiber. This can be achieved by increasing the bandwidth in the time domain, the frequency domain, and the spatial domain. The time domain refers to the modulation rate, or symbol rate, or baud rate^1^. In the spectral domain, several spectrally narrow lines can be used for independent data transmission, which is referred to as wavelength-division multiplexing (WDM)^2,3^. The spatial domain refers to the use of multicore fibers^4,5^. In the simplest case, bits are encoded in a non-return-to-zero (NRZ) format, where light intensity has two states, 0 or 1. Bit rate can be increased by using more advanced intensity modulation direct detect (IM/DD) formats, such as PAM*m* (pulse amplitude modulation with *m* intensity levels), which encode $\log _2(m)$ bits per symbol, or by using coherent QAM formats that modulate both the intensity and the phase of the electromagnetic field^6^. In practice, interconnects mostly use NRZ (especially for long reach $>30$ km) and PAM4 (mainly for shorter reach $<30$ km) formats, which are technically less challenging and more robust with respect to different noise sources. Also, at present, single mode single core fibers are mostly used in practical networks. On the other hand, WDM is already routinely used in conventional networks. In the O-band, the standard wavelength grids are CWDM, with 4 wavelengths separated by about 20 nm, and LWDM, having 4 or 8 lines with 4.5 nm (800 GHz) separation. A further increase in the bandwidth implies using a dense WDM (DWDM) grid.

Currently, different wavelengths for WDM are provided by distributed feedback (DFB) lasers operating at different wavelengths. DFB lasers provide enough power to be used without an amplifier and have very low relative intensity noise (RIN). However, each laser needs to be independently coupled to the photonic integrated circuit (PIC) where the light is modulated and multiplexed. Coupling with submicron accuracy constitutes the main cost to the transceiver which, will scale with the increasing number of channels. The other approach is using an array of DFB lasers processed with a well-defined pitch on the same chip. It simplifies the coupling task, but brings other challenges related to the limited tolerance of the DFB wavelength and mainly to the yield of valid lasers. If we consider a high yield for a single DFB of 0.9, then the yield for an array of 16 such DFBs will be $0.9^{16} \approx 0.2$. Therefore, it is tempting to have a single laser source with multiple wavelengths.

These sources are known as comb lasers and have multiple implementations^7^ and applications^8–11^. The two dominating technologies are Kerr combs (employing either supercontinuum generation in waveguides^12^ or nonlinearities in microresonators^13^) and semiconductor mode-locked lasers (MLL)^14–16^. The Kerr comb has the advantage that its interline separation can be easily tailored and can cover a wide spectral range^5,17–19^. At the same time, the Kerr comb has a relatively low power efficiency of 1–3%^17,19,20^ compared to semiconductor MLL. Also it is hard to make this solution monolithic.

Semiconductor mode-locked comb lasers are Fabry–Perot lasers with mirrors formed by the cleaved surfaces of the chip with deposited dielectric layers. Their active medium is formed of quantum dots (QDs)^14–16^ or quantum dashes^21–23^. The spectrum of such lasers shows a number of longitudinal modes with separation defined by the cavity length *L*, $\Delta \nu = c / (2 n_\text {gr} L)$, where *c* is the speed of light, and $n_\text {gr}$ is the group refractive index. Usually, competition between modes in Fabry-Perot lasers leads to a large RIN of individual modes. However, in the mode-locking regime, modes are phase-locked to each other, which dramatically decreases the RIN of individual modes and makes them suitable for data communication. Furthermore, typically, passive mode locking in lasers is associated with the formation of pulses to minimize the absorption^24,25^. In QD lasers, in fact, mode locking may take place without pulse formation^14^ which stimulates debates about the fundamental origin of mode locking in this system^26,27^ and makes this system more suitable for data transmission, minimizing nonlinear effects in the PIC. This is the so-called frequency-modulation mode locking (FM-ML) in contrast to the amplitude-modulation mode locking (AM-ML).

Several works analyse the application of comb sources for data transmission in both the O-band^28–31^ and the C-band^5,32–36^. The studies in the C-band mostly deal with coherent QAM formats underlying long-reach interconnects. On the other hand, the O-band studies are performed mostly for IM/DD formats, while the work^31^ covers both approaches in the O-band. Research on DWDM data transmission mostly focuses on maximizing the total bandwidth by increasing the spectral density of the data. Namely, this is accomplished by increasing the number of channels while making the interline separation relatively low—about the baud rate. In practice, however, several limitations come into play. The number of channels is limited by the total power delivered to the PIC, in order to prevent its damage, and by the noise within each channel at the photodetector, which sets the lower bound on the power of a single channel. Thus, each channel should provide enough power for reliable detection, but all channels combined must not damage the PIC with their total power. This hinders the achievement of a data stream involving all usable comb lines simultaneously, and, to the best of our knowledge, so far in the O-band, data transmission was demonstrated only for the comb lines selected one by one on the transmitter level.

The other challenge is the amplification of the signal to compensate for the large losses usually associated with the PIC. First, any amplifier will induce additional noise associated with amplified spontaneous emission (ASE) and gain saturation^37^. Second, ideally, one amplifier should amplify all channels simultaneously after the PIC, while being a compact device compatible with the pluggable transceiver form factor. So far, amplification was accomplished using bulky PDFA or BDFA in the O-band or EDFA in the C-band, while in the work^38^ it was shown that QD SOAs can be superior to PDFA in the O-band.

And finally, the interline separation should not be too small to avoid crosstalk^39^ and to be supported by the state-of-the-art PIC technologies compatible with mass production. Note that so far the smallest interline separation available on the mass market is 800 GHz (LWDM) and it is hard to expect the rapid support of an interline separation below 100–200 GHz.

In this work, we demonstrate a compact and energy-efficient solution for the O-band comprising a semiconductor QD comb laser with a 100 GHz interline separation and a QD semiconductor optical amplifier (SOA) both in a compact chip design. The comb laser supports a tunable number of low-noise channels ranging from 10 to 23, which can be controlled by the voltage on the saturable absorber section. Thus, the width of the comb spectrum with line intensities within the 3 dB interval is as high as 2.3 THz. We analyse the noise of the comb by measuring the RIN and bit-error rate (BER) of separate lines and observe a strong correlation between the noise and the intensity of the line. We demonstrate data transmission of a PAM4 signal with a total bit rate in a fiber of up to 2.3 Tb/s under the HD-FEC limit thanks to amplification of all modulated channels by a single SOA. Furthermore, we present preliminary results on the development of the QD mode-locked comb lasers with interline separation increased up to 216 GHz with chip lengths down to 200 $\upmu$m.

## Laser structure and experimental details

**Fig. 1:**
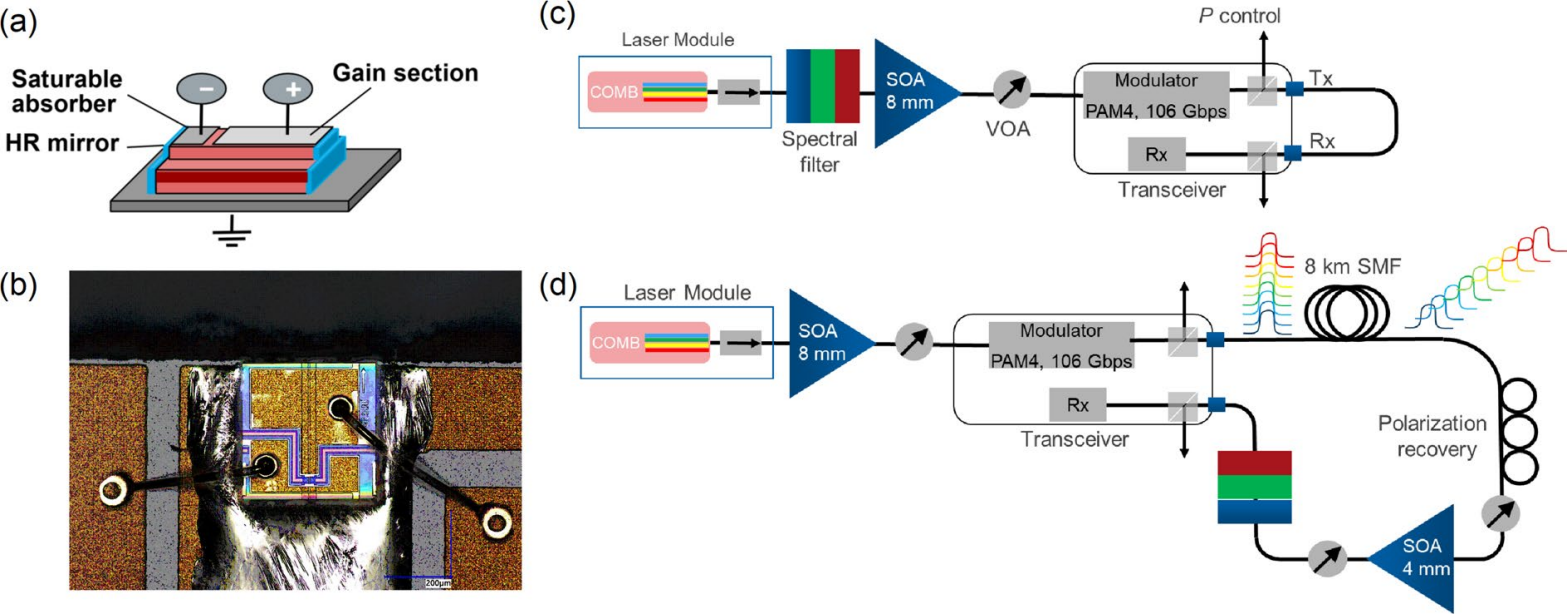
(**a**) Scheme of DWDM comb laser source: the cavity is formed by the etched mesa and mirrors on the facets, while the active medium is formed by the epitaxial QD layers. Stable mode locking is ensured by the saturable absorption section, electrically isolated from the gain section. (**b**) Top view photo of the real 100 GHz comb laser device. Its dimensions are $0.4 \times 0.4$ mm$^2$. (**c**) Experimental scheme for measuring the BER of separate comb lines. (**d**) Experimental scheme for amplification of the modulated signal of all comb lines via a single SOA and measuring their BER.

### Comb laser

The comb laser source (Fig. 1a) produced by Innolume GmbH is a ridge laser with a shallow mesa. It is grown by Molecular Beam Epitaxy on GaAs *n*-type substrates with InGaAs/GaAs QDs as the active medium. The vertical profile of the laser mode is defined by the epitaxial layers, while the horizontal mode profile is shaped by the etched mesa. A voltage applied between the *p*-doped mesa and the *n*-doped substrate results in a current leading to recombination of the carriers inside the QDs. The laser cavity is formed by cleaved facets, whose reflections are tailored by appropriate dielectric coating. The laser spectrum shows a number of longitudinal modes, whose separation is defined by the cavity length. To ensure stable mode locking, we use an integrated absorber section, which is electrically isolated from the gain section and therefore can be biased separately. The main results of the article are for the comb laser with an absorber section and a total cavity length of 407 $\upmu$m, providing an interline separation of 100 GHz. Its top view is shown in Fig. 1b. The laser chips are packaged into pigtailed 14-pin butterfly modules, which allow temperature stabilization. The laser output beam is collimated by free-space fast and slow axis collimators and directed to a polarization-maintaining fiber through a double-stage optical isolator. The total coupling efficiency into the fiber is 57%. We also present the results for the 250 $\upmu$m device with an absorber section and 295 and 188 $\upmu$m devices without an absorber section, all three are unpackaged. Emission of the unpackaged devices is also collimated by a lens, transmitted through a free-space optical isolator, and coupled to a fiber using another lens.

### Semiconductor optical amplifier

To amplify the comb laser emission before and after modulation, we use QD-based high-saturation-power SOAs produced by Innolume with 8 and 4 mm chip lengths, respectively. The SOAs have a noise figure of 5 dB, a saturation output power of about 20 dBm, and a small-signal gain of more than 20 dB (10 dB) for the 8 mm (4 mm) chip, depending on the operating current. The SOAs have a gain spectrum with the central wavelength tuned by temperature in the vicinity of 1300 nm and a bandwidth of about 30 nm. The SOAs are packaged in 14-pin fiber-coupled butterfly modules with a thermoelectric cooler/heater. More details on these SOAs, including their ability of data transmission, are given in Ref. ^37^.

### Experimental

**Fig. 2:**
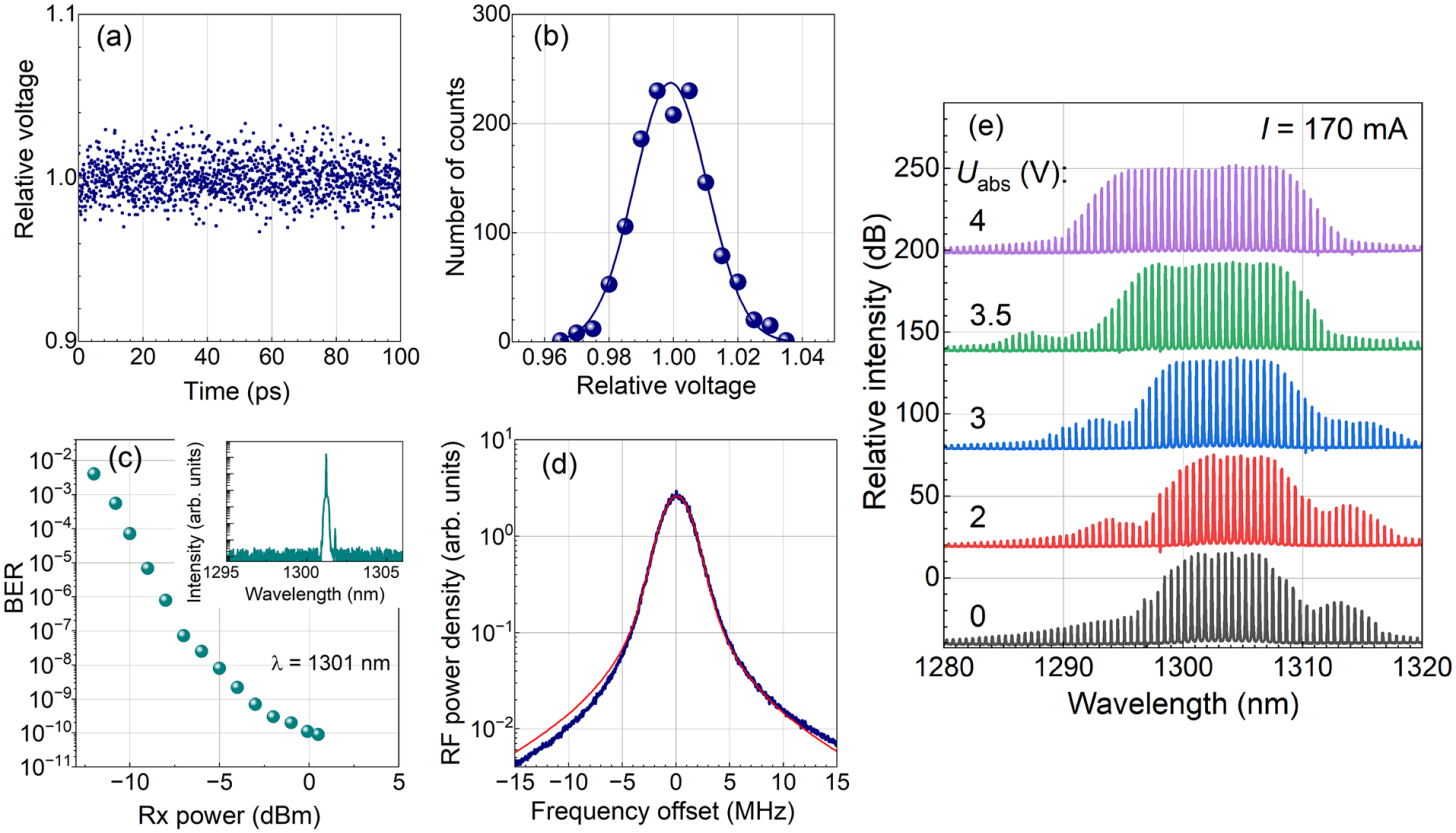
(**a**) Oscilloscope trace showing the signal from the photodetector registering the comb line at 1301 nm. The spread of the noise gives a measure of the RIN. $U_\text {abs} = 0$. (**b**) Distribution of the oscilloscope signal fitted with a Gaussian function. $U_\text {abs} = 0$. (**c**) Waterfall curve showing the BER as a function of the registered optical power for the selected comb line, whose spectrum is shown in the inset. $U_\text {abs} = 0$. (**d**) Self-heterodyne radiofrequency (RF) spectrum of the comb line at 1301 nm (blue line) fitted with the Voigt function (red line). $U_\text {abs} = 0$. (**e**) Spectra of the 100 GHz comb laser at different reverse biases $U_\text {abs}$ at the saturable absorber. The comb laser is operated at $T = 25 \; ^{\circ }$C and a current of 170 mA (**a**–**c**,**e**) and 157 mA (**d**).

We measure the optical spectra of the lasers using an Optical Spectrum Analyzer (OSA from Yokogawa). To measure the RIN, we filter out a separate comb line, amplify it with an SOA, and register its intensity with a linear photodetector having a bandwidth of $\Delta f = 10$ GHz, connected to the oscilloscope. We have verified that in the considered measurement regime, the SOA contribution to the RIN is small. The typical oscilloscope trace showing the intensity noise is presented in Fig. 2a. The distribution of the photodiode signal is shown in Fig. 2b. The distribution is fitted with a Gaussian function, from which, the intensity variance $\sigma$ is determined. RIN in dB/Hz is calculated as $10 \log _{10}((\sigma /\mu )^2 / \Delta f)$, where $\mu$ is the average signal intensity.

To test the data transmission with separate comb lines, the scheme shown in Fig. 1c is used. A comb line is spectrally filtered and amplified using an SOA module with an 8 mm cavity length operated at a temperature of 40 $^\circ$C and a current of 2.5 A, resulting in a gain of about 27 dB to compensate for the losses in the transceiver. The power of the line is adjusted by a variable optical attenuator (VOA) to guarantee the same power for all lines at the photodetector. The laser line emission is directed to the custom-modified transceiver, with internal lasers replaced by fiber outputs delivering the emission of an external (comb) laser.

The PIC inside the transceiver provides 106 Gb/s PAM4 modulation of the input emission with a PRBS31 sequence generated internally in the transceiver. The emission from the transmitter (Tx) port of the transceiver is directed right away to the receiver port (Rx) of the same transceiver, where the light is detected, the PRBS31 sequence is analysed, using an internal analyser, and the BER is calculated. Very similar BER values are registered when an external generator and an analyzer on the evaluation board are used for generation and analysis of the PRBS sequence.

The spectrum of the filtered and modulated comb line is shown in the inset of Fig. 2c, while the dependence of the BER on the registered optical power (ROP)—the waterfall curve—is shown in the main panel of Fig. 2c. In what follows, we measure the BER for all lines within the same use case at the same ROP to ensure that BER variations are not influenced by the photodetector. The measurements for the 100 GHz comb laser at 0 voltage at the saturable absorber are performed at an ROP of 0 dBm, while all other measurements are performed at an ROP of -3 dBm.

To demonstrate the simultaneous data transmission of multiple laser lines and the amplification of the optical signal, we use the scheme depicted in Fig. 1d. Here, all lines are simultaneously amplified using an 8 mm SOA operated at 1.5 A and 40 $^\circ$C and providing a gain of about 10 dB (in the saturation regime due to the high input power of the total comb emission). The amplified emission is modulated at 106 Gb/s PAM4. The modulated signal from the Tx port is transmitted through 8 km of single-mode optical fiber to induce a time delay between symbols at different wavelengths (thanks to fiber dispersion) and mimic independent data sequences in different channels. We note that although all channels carry the same information, any two consecutive bits in the PRBS sequence are quasi-independent. Therefore, after the delay in the fiber, we have quasi independent bits in different channels at every given time moment. In this way we perform a laboratory emulation based on fiber-dispersion-induced decorrelation, while all channels still share the same electrical modulation source. Then the polarization of the light is recovered, and the signal is amplified by the 4-mm low-noise SOA operated at 1 A and 40 $^\circ$C and providing a 14 dB gain. The dependence/independence of bits in different channels may play a role for the pattern effect in this SOA, taking place at high input powers^37^, which is not the case in our experiment (see below). To analyse the BER, separate lines are filtered out one by one and detected by the Rx of the same transceiver.

## Characterisation of the comb laser

**Fig. 3:**
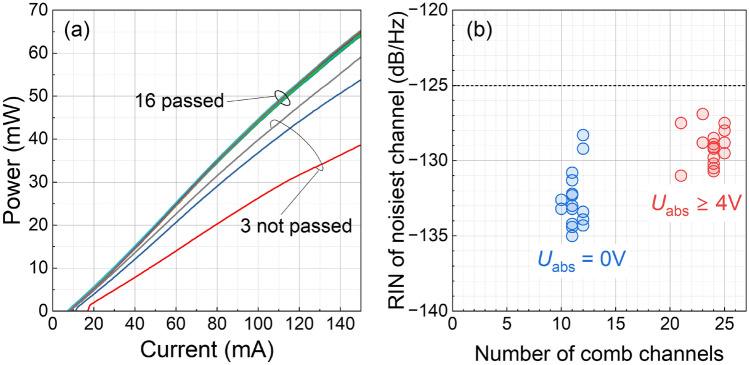
Comb laser statistics. (**a**) Total comb laser intensity versus current (LI curves) for 19 tested chips. The saturable absorber section is disconnected. (**b**) RIN of the noisiest mode (averaged over a 10 GHz interval) for 16 selected lasers versus the number of comb lines within the 3 dB intensity interval. The data are shown for zero and high saturable absorber voltages at a laser current of 200 mA. Laser temperature in (**a**) and (**b**) is 25 $^\circ$C.

The spectra of the semiconductor QD comb laser at different reverse bias voltages at the saturable absorber section $U_\text {abs}$ are presented in Fig. 2e. The spectrum consists of a number of equidistant (in the frequency domain) longitudinal modes. The shape of the spectrum at low $U_\text {abs}$ is characterized by a ”flat top” with 11 modes within the 3 dB intensity interval and side maxima presumably originating from the four-wave mixing of the main modes. The total intensity of the laser is 74 mW from the chip and 42 mW from the fiber, corresponding to about 6.0 and 3.4 mW per mode, respectively. Here, we take into account that useful modes (with intensities within the 3 dB interval) provide about 90% of the total power. As the voltage $U_\text {abs}$ is increased, the spectrum broadens, reaching 23 modes within the 3 dB intensity interval at $U_\text {abs} = 4$ V. Here, the total intensity is 60 mW from the chip and 34 mW from the fiber, corresponding to 2.3 and 1.3 mW, respectively, per mode. Taking into account that the measurements were performed at a laser current of 170 mA and a voltage of 2.1 V, the power conversion efficiency exceeds 15% and 8% for the useful power from the chip and from the fiber, respectively.

The mode-locking regime of the comb laser is evidenced by the low RIN and low BER of separate comb lines (Fig. 2a–c and the following results). The other indication of mode locking is the narrow linewidth of the comb modes. The linewidth of every mode is too narrow to be resolved by an OSA. It is measured using the self-heterodyne technique^40^, where the convolution of the delayed emission of the selected comb line with the modulated emission of the same line is detected using a photodiode and an electrical spectrum analyzer. In this way, the spectrum is transferred from the optical domain to the radiofrequency (RF) domain. The details of our experimental setup can be found in Refs. ^15,41^. Figure 2d shows the RF power spectrum of a single comb line at 1301 nm. This spectrum gives the total optical linewidth of a single comb line of 1.6 MHz. The spectrum is fitted with a Voigt function, taking into account the inhomogeneous (Gaussian) and homogeneous (Lorentzian) components. The homogeneous component of the spectrum is usually associated with the phase noise, and the sub-megahertz width of the Lorentzian component is a strong indication of the mode-locking regime. We have verified that in the considered comb structure with 100 GHz interline spacing, stable mode locking is observed for pumping currents from 150 to 250 mA and for $U_\text {abs}$ from 0 to 5 V at a device temperature of $25 \; ^{\circ }$C.

The presented comb device gives a record-high number of 23 100-GHz-separated modes with intensities falling within the 3 dB interval. It is interesting to verify the repeatability of this result. We test 19 chips by measuring the optical power as a function of the applied current (Fig. 3a). Sixteen chips show power spreads of less than 3%, while the other three show considerable deviations. From the 16 chips with the highest output powers, we assembled butterfly modules and tested them for (i) the number of modes with intensities within a 3-dB interval and (ii) the RIN of these modes, averaged over a 10-GHz bandwidth. In Fig. 3b we plot the RIN of the noisiest mode (i.e. maximal RIN) versus the number of modes. Each point corresponds to one laser. The data are presented for zero and high voltages at the saturable absorber. One can see that all lasers show RIN of their modes below $-125$ dB/Hz indicating the mode-locking regime for all lasers. The number of modes for $U_\text {abs} = 0$ V is 11 for 9 lasers, 12 for 5 lasers, and 10 for 2 lasers. The number of modes achieved for $U_\text {abs} \ge 4.0$ V is 24 for the majority of lasers with variation from 21 to 25. This means that for all investigated lasers, by tuning $U_\text {abs}$, it is possible to achieve 21 low-noise comb modes with intensities within the 3 dB interval, which is still well beyond previously reported results for 100 GHz MLL.

## Noise of the comb modes, data transmission

**Fig. 4:**
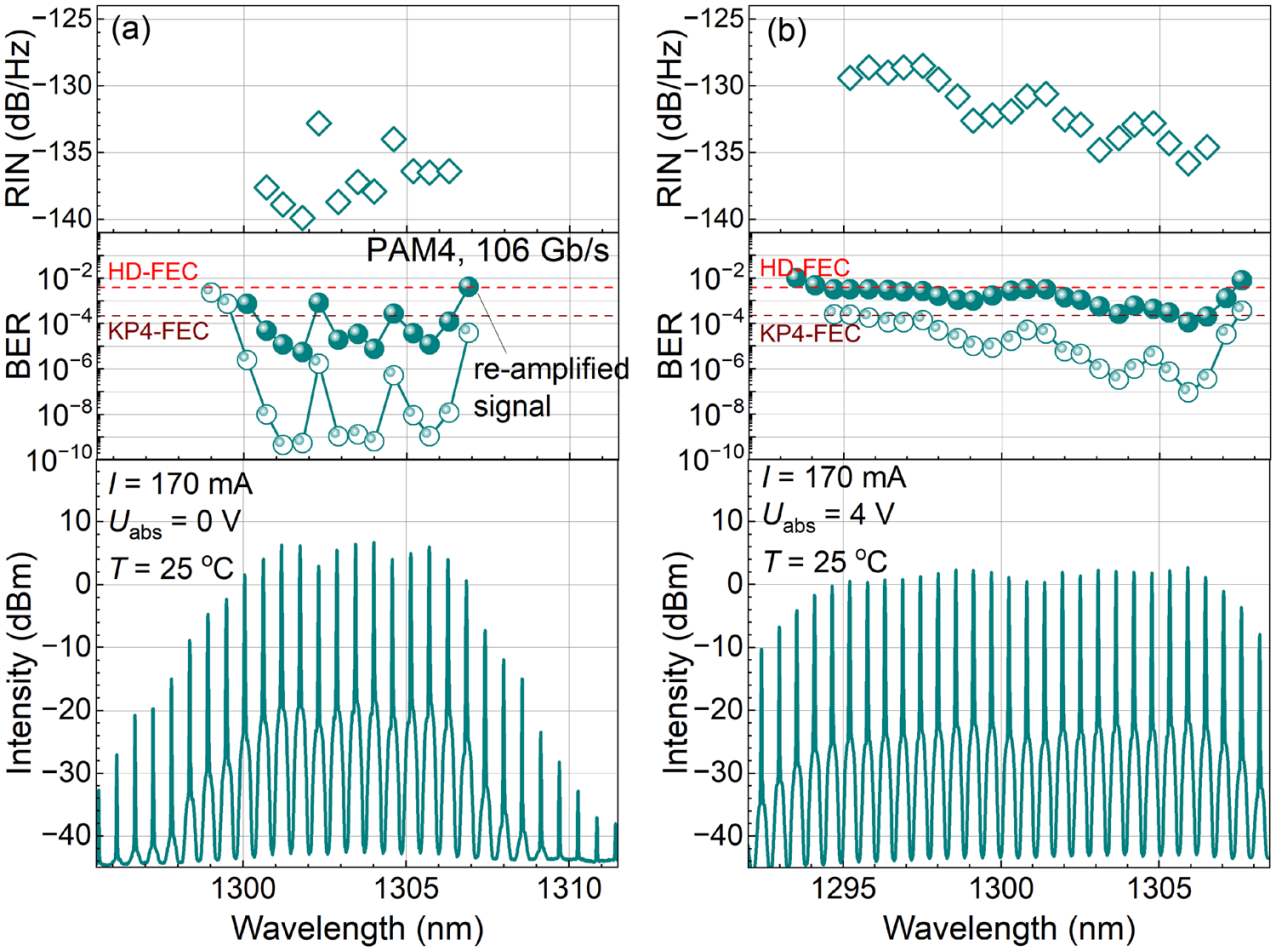
Spectra of the 100 GHz comb laser after amplification and modulation (bottom panel). The middle panel shows the BER for separate comb lines used for data transmission of a 106 Gb/s PAM4 signal without (open symbols) and with (full symbols) amplification after modulation. The upper panel shows the RIN of separate lines averaged over a 10 GHz range. Panel (**a**) corresponds to a 0 V bias voltage on the saturable absorber, (**b**) corresponds to 4 V. The laser current is 170 mA, and the temperature is $25\; ^\circ$C.

Next, we select a separate mode using a spectral filter and measure its RIN. Also, we use separate modes for the data transmission of a 106 Gb/s PAM4 signal and measure the corresponding BER. Figure 4a shows the spectrum of the comb laser at $U_\text {abs} = 0$ V after PAM4 modulation (bottom panel) together with the RIN (top panel) and the BER (open symbols in the middle panel) of the main modes as a function of the wavelength. The RIN of 9 modes is below −135 dB/Hz, while for 2 middle modes it is between −135 and −130 dB/Hz. At the same time, the BER for 9 modes is $\lesssim 10^{-8}$, while for the same 2 modes the BER is about $10^{-6}$. We note that for 13 modes the BER is lower than $2.2 \times 10^{-4}$, the KP4-FEC limit, where errors can be safely corrected. It is worth mentioning that the two modes with increased RIN and BER have lower intensity compared to the other main modes.

At higher $U_\text {abs} = 4$ V, 23 main modes can be identified (Fig. 4b). The RIN for these modes is higher than for the case of $U_\text {abs} = 0$ V, ranging from −136 to −127 dB/Hz. The BER is also higher, but stays below or about the KP4-FEC limit. Note that both RIN and BER tend to decrease with the wavelength, correlating with the slight increase of the mode intensity. We remind that the ROP at the photodetector is kept constant across different lines, thus the increase in RIN and BER with the decrease of the laser mode intensity is not related to the finite sensitivity of the photodetector.

From Fig. 4 we see that BER correlates with the RIN and that both correlate with the mode intensity. We analyse this more quantitatively using the optical signal-to-noise ratio (OSNR) $Q = P / \sigma$, where *P* is the optical power of the mode and $\sigma$ is the noise distribution variance. *Q* can be estimated both from RIN and from BER:

1 $$\begin{aligned} Q_\text {RIN} = (\text {RIN} \cdot \Delta f)^{-1/2},\end{aligned}$$

2 $$\begin{aligned} Q_\text {BER} = 6 \sqrt{2} \cdot \text {erfc}^{-1}(8 \cdot \text {BER}), \end{aligned}$$

where $\text {erfc}()$ is the complementary error function. The formula for $Q_\text {RIN}$ is straightforward, where we take $\Delta f = 10$ GHz, the interval in which the RIN is averaged. For $Q_\text {BER}$ we take into account that the data transmission is in the PAM4 format and that the highest intensity level has the largest noise^37^. So we assume that all errors come from Gaussian noise at the highest intensity level and that the decision threshold is in the middle between the levels.

We note that the BER is influenced by the laser RIN and also mostly by the sensitivity of the photodetector. For a Gaussian distribution of the noise, the squares of the noise amplitudes ($\propto 1/Q^2$) from different contributions add up. Thus, we expect that

3 $$\begin{aligned} \frac{1}{Q_\text {BER}^2} = \frac{1}{Q_\text {PD}^2} + \frac{1}{Q_\text {RIN}^2}. \end{aligned}$$

**Fig. 5:**
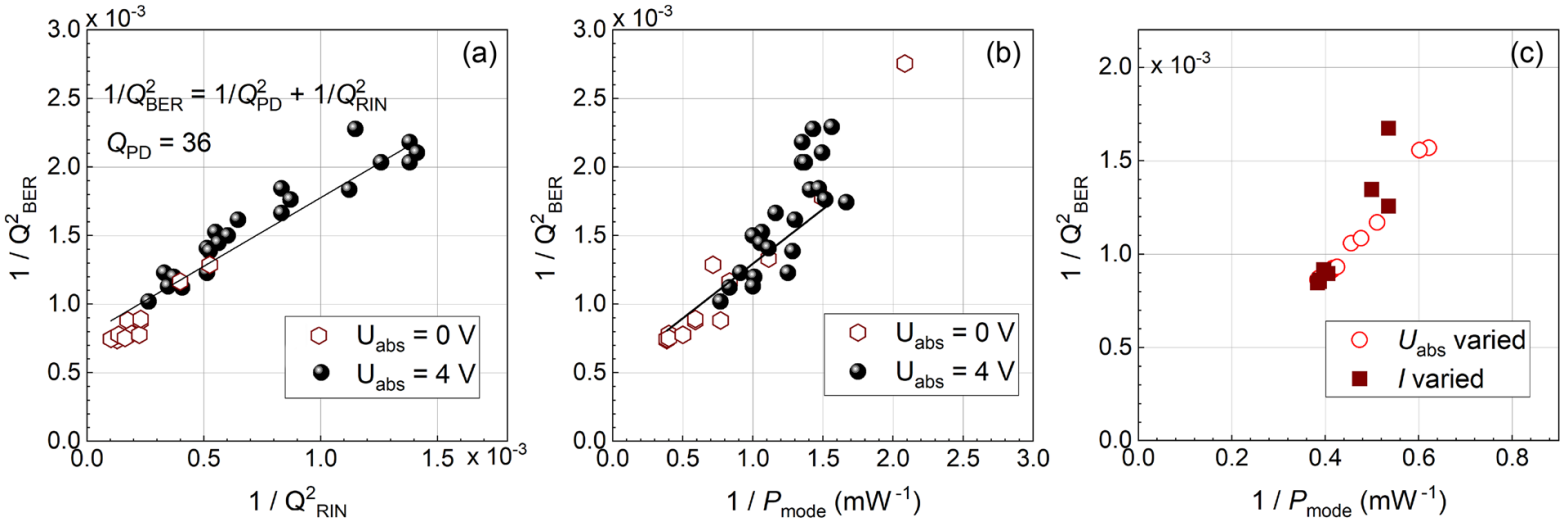
(**a**) Correlation of the *Q* factors extracted from RIN and BER: dependence of $1/Q_\text {BER}^2$ on $1/Q_\text {RIN}^2$. (**b**,**c**) Correlation of BER and the mode power: dependence of $1/Q_\text {BER}^2$ on $1/P_\text {mode}$. Different points in (**a**) and (**b**) correspond to different comb lines for $U_\text {abs} = 0$ (open symbols) and $U_\text {abs} = 4$ V (closed symbols), the laser current is 170 mA. Different points in panel (c) correspond to variation of $U_\text {abs}$ for $I = 170$ mA and variation of *I* for $U_\text {abs} = 0$.

To check this expectation, we plot $1/Q_\text {BER}^2$ as a function of $1/Q_\text {RIN}^2$ in Fig. 5a, where different points correspond to different modes, both for $U_\text {abs} = 0$ V and $U_\text {abs} = 4$ V. We see a clear dependence between the two quantities, which is close to linear, as expected from Eq. (3), and indicates the Gaussian distribution of the noise. Nevertheless, the slope of the dependence is about 1.5, which is related to the assumptions taken when determining the *Q* factor.

Next, we check the observed correlation between the noise measured via BER and the intensity of the mode. First, we plot $1/Q^2_\text {BER}$ versus $1/P_\text {mode}$ in Fig. 5b for different modes at $U_\text {abs} = 0$ V and $U_\text {abs} = 4$ V. Then, in Fig. 5c we plot $1/Q^2_\text {BER}$ versus $1/P_\text {mode}$ for a single mode at different values of the laser current and $U_\text {abs}$. Note that the power at the photodetector is kept constant also in this experiment. We see that in both cases there is a clear dependence, which is close to linear. Such a behaviour is characteristic for the noise arising from the interference of the laser mode with ASE, so-called beat noise^37^.

## Practical scheme for DWDM data transmission link using comb laser and SOA

**Fig. 6:**
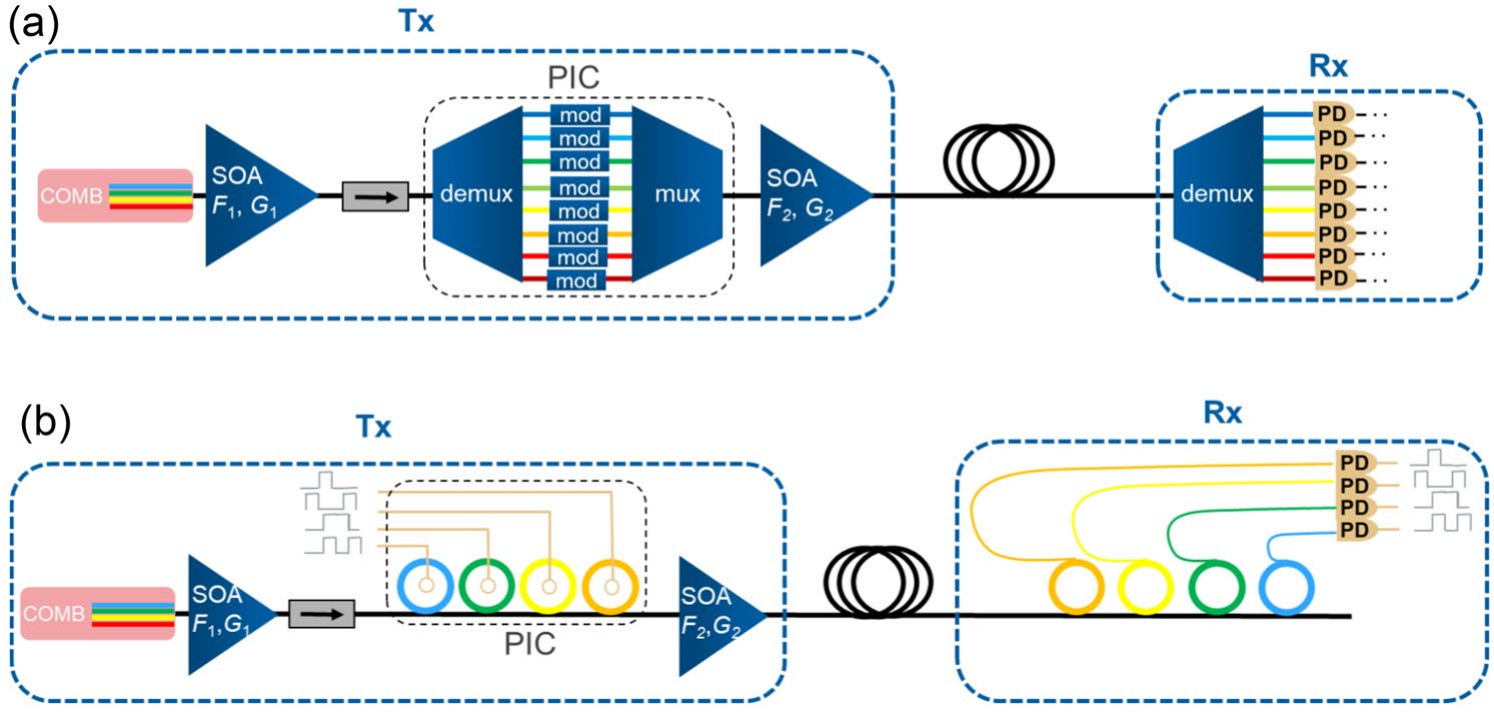
Proposed practical implementation of comb-based DWDM data transmission with amplification of the modulated WDM signal. (**a**) Scheme with a separate demultiplexer, multiplexer, and modulators. (**b**) Scheme with ring modulators combining multiplexing and demultiplexing.

**Table 1:** Comb-SOA-based transmitter use cases.

#	Use case	$P_\text {mode}$ (dBm)	$N_\text {modes}$	$G_1$,$F_1$ (dB)	PIC loss (dB)	$G_2$,$F_2$ (dBm)	Link loss (dB)	$P_\text {mode}$at PD (dBm)	Bit rate (Tb/s)	BER, PAM4, meas.	BER, NRZ, calc.
1	comb-SOA-PIC-SOA,$U_\text {abs} = 0$	5.2	11	10, 5	−23	14, 5	−6	0	0.9 (KP4-FEC)	$10^{-5}$	$<10^{-13}$
2	comb-SOA-PIC-SOA,$U_\text {abs} = 4$	0.9	23	10, 5	−23	14, 5	−5	−3	2.3 (HD-FEC)	$10^{-3}$	$<10^{-13}$

A proposed simplified practical implementation of the comb-based transmitter is shown in Fig. 6. Here, the PIC can involve a demultiplexer, modulators and a multiplexor (Fig. 6a). But an alternative and even more practical solution, for a PIC in a DWDM transmitter is an array of ring resonators, which can selectively modulate separate comb lines (Fig. 6b)^42–45^. The comb emission can be amplified by using an SOA to compensate for large losses in the PIC including the finite coupling efficiencies to and from the PIC. The total losses can easily reach −20 dB^44^. The amplification of the comb emission can take place before the PIC, after the PIC, and in both instances. We note that the total power incident to the PIC is limited to a few hundred milliwatts due to nonlinear effects in the PIC leading to its overheating and damage. This requires the use of an SOA after the PIC to ensure enough power at the photodetector. Thus, the scheme without an SOA after the PIC is impractical. Both schemes with and without an SOA before the PIC can be used. The scheme without the first SOA has the advantage of simpler implementation, less strict requirements for the optical isolator before the PIC, and less power consumption. Its disadvantage is higher noise, which increases the BER for PAM4, but should still provide error-free operation for NRZ. The scheme with the first SOA requires more advanced integration, consumes more power, but ensures lower BER.

We demonstrate the implementation of the comb-SOA-PIC-SOA scheme using the experimental setup shown in Fig. 1d with an “effective” PIC comprising a modulator and an 8-km-fiber to delay and decorrelate bit sequences in different channels. The results of BER measurements for each comb line, after transmitting the modulated signal through the second SOA, are shown by full symbols in the middle panels of Fig. 4. Expectedly, the BER is higher than in the experiment with the single comb lines, as the second SOA introduces noise. In the single line experiments every comb line is amplified to $\sim 100$ mW before entering the PIC which allows data transmission with one line at a time, while several lines with such power would be unacceptable for the PIC. In the experiment with simultaneous data transmission on multiple lines (Fig. 1d) the incident power of each line entering the PIC didn’t exceed 10–30 mW which required the use of the second SOA after PIC. In this way, data transmission of a PAM4 signal with simultaneous multiline amplification using one SOA is possible with BER below the KP4-FEC limit for 9 lines and with BER below the HD-FEC limit for 23 lines. These two cases correspond to a total bit rate (below mentioned above FEC limits) of about 0.9 and 2.3 Tb/s.

Since the presence of the second SOA after modulation is detrimental for multiline data transmission, it is worth explaining its effect on the BER. The SOA-induced BER degradation is caused by the beat noise resulting from SOA ASE and by the pattern effect resulting from gain saturation^37^. We can quantify these contributions by the corresponding optical signal-to-noise-ratio *Q*. For the beat noise, $Q_\text {bn} = \sqrt{P_\text {in}/2h\nu F R_\text {tot}}$, while for the gain saturation effect, $Q_\text {gs} = \sqrt{9 \tau R_\text {tot} /20}(P_\text {in} + P_\text {sat,in}) / P_\text {in}$^37^. Here $h\nu = 0.95$ eV is the photon energy, $F \approx 5$ dB is the SOA noise figure, $\tau \approx 150$ ps is the gain recovery time, $P_\text {in,sat} \approx 10$ dBm is the SOA saturation input power, $P_\text {in} \approx 0$ dBm is the total SOA input power, $R_\text {tot} = 1.1$ (2.3) Tb/s is the total bit rate for $U_\text {abs} = 0$ V (4 V). For the case of $U_\text {abs} = 0$ V (4 V) we can estimate $Q_\text {bn} = 32$ (21), $Q_\text {gs} = 84$ (126). Thus, the BER degradation is dominated by the beat noise. The SOA-related *Q* factor contributes to the total *Q* factor of the system by another term in Eq. (3). From the *Q* factor of the system without the second SOA (Fig. 5) of $\sim 35$ (25) for $U_\text {abs} = 0$ V (4 V) we can estimate the *Q* factor of the system with the second SOA of 23 (16) and corresponding BER of $2 \times 10^{-5}$ ($0.8 \times 10^{-3}$). These BER values are close to those obtained experimentally in Fig. 4. We note that the key parameter of an SOA, controlling its beat noise and, thus, BER degradation in the considered regime, is the noise figure. It should be kept as low as possible.

In the previous section, we showed that the measured level of BER corresponds to the noise determined from the RIN measurements, which implies a Gaussian noise distribution. The Gaussian distribution of the noise allows us to evaluate the expected BER for NRZ using the measured BER for PAM4 with the same 106 Gb/s bit rate per line. In the simplified picture, BER for NRZ is about $\text {erfc}(Q/\sqrt{2})$, while for PAM4 it is about $\text {erfc}(Q/3\sqrt{2})$^37^. The estimated NRZ BER is below $10^{-13}$ for the considered two experimental cases, ensuring error-free data transmission. The summary of the demonstrated use cases is presented in Table 1. It shows the parameters of the comb, SOAs, PIC losses, ROP, and BER for data transmission with PAM4 (measured) and NRZ (calculated) 106 Gb/s/line data signals.

## Towards higher interline separation

**Fig. 7:**
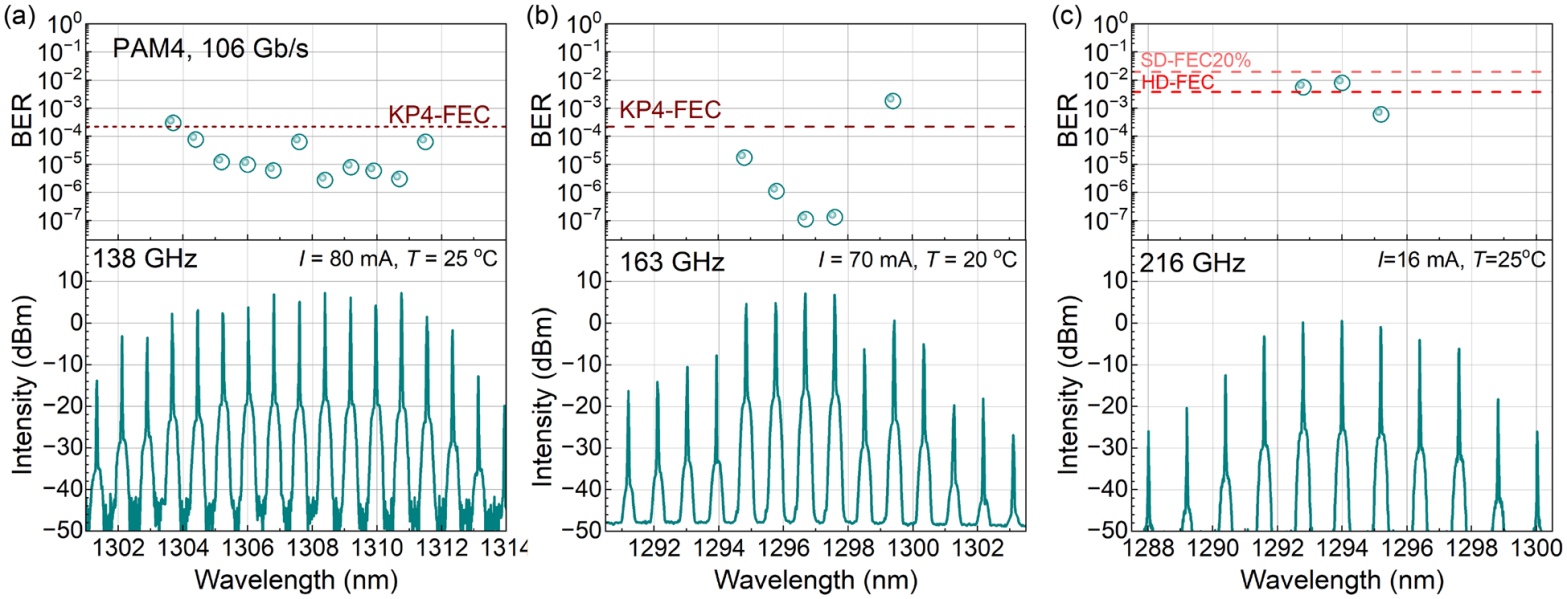
Spectra of the comb lasers after amplification and modulation (bottom panel). The upper panel shows the BER for separate comb lines used for data transmission of the 106 Gb/s PAM4 signal. Panels (**a**–**c**) correspond to comb lasers with cavity lengths of 295 $\upmu$m (**a**), 250 $\upmu$m (**b**), and 188 $\upmu$m (**c**), corresponding to intermode separations of 138, 163, and 216 GHz, respectively.

**Fig. 8:**
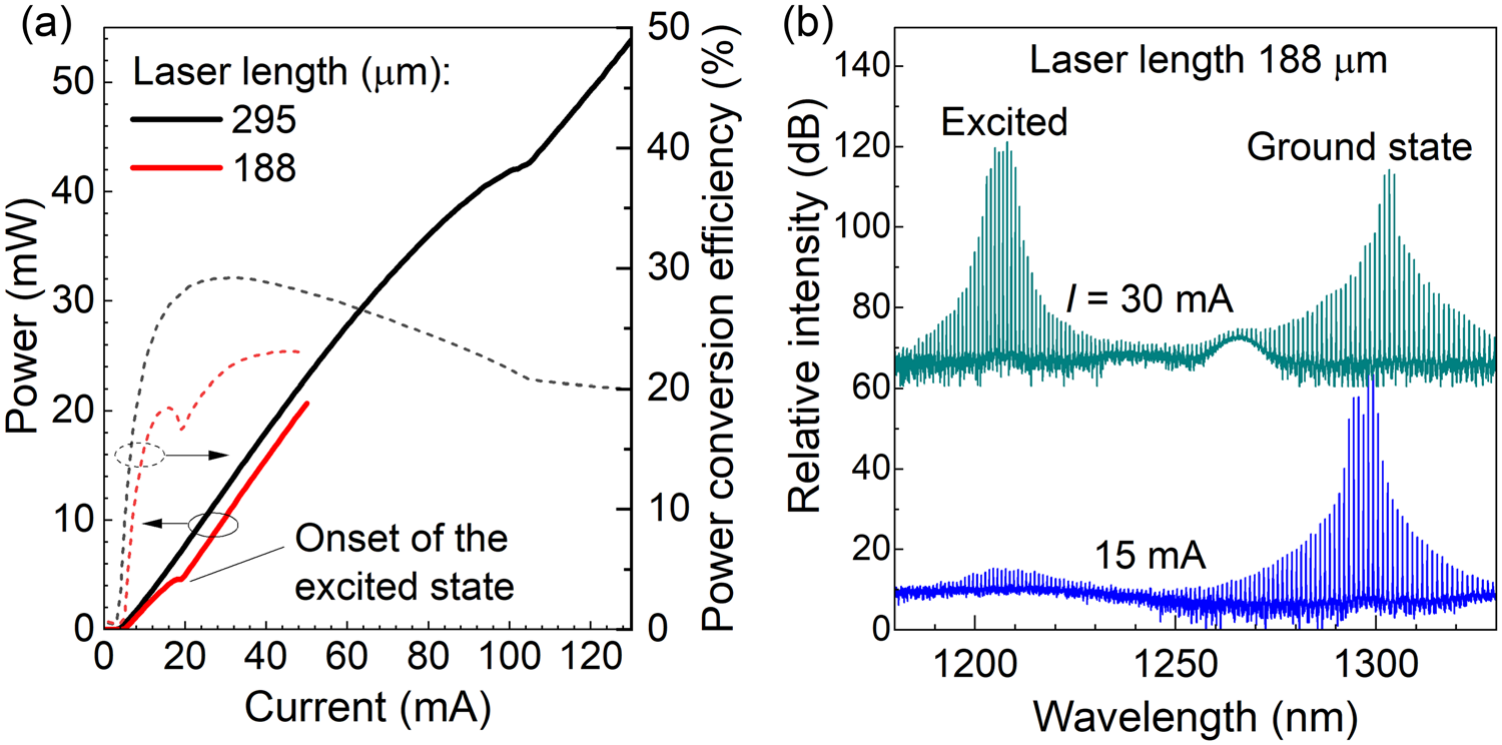
(**a**) Laser power (left axis) and PCE (right axis) as a function of the current for lasers formed from the same structure and having different cavity lengths. The lasers have one high-reflection (HR) mirror, while the other mirror is formed by an as-cleaved facet. (**b**) Spectra of the shortest laser at different currents showing the onset of the excited state. The lasers are kept at a temperature of 25 $^\circ$C.

We have demonstrated the feasibility of data transmission using a single DWDM 100 GHz comb source, which has a number of advantages compared to using multiple DFB lasers in WDM data transmission. One of the main technical difficulties preventing the immediate use of this concept is the relatively small interline separation (100 GHz) requiring more advanced demultiplexing techniques. While silicon photonics technology is on the way to making small interline separation schemes more affordable, we approach the requirements of modern PICs by increasing the interline separation of the comb lasers. Figure 7 shows the spectra and BER of single lines for the comb devices having an interline separation of 138, 163, and 216 GHz, respectively. The increased interline separation is achieved by reducing the laser length to 295, 250, and 188 $\upmu$m, respectively. Note that devices with 295 and 188 $\upmu$m are implemented without a saturable absorber section. While the 250 $\upmu$m device has a saturable absorber, the bias voltage has the same polarity as for the main section. This provides larger total gain, but results in a limited range of currents where stable mode locking is observed, as well as an increased sensitivity of the lasers to optical feedback. These experiments also show that mode locking in QD lasers can be achieved without a saturable absorber section. The latter makes mode locking more stable with respect to perturbations (such as optical feedback). Interestingly, for the device with an interline separation of 138 GHz (Fig. 7a) we observe 10 low-noise lines having a power of about 3.6 mW per line (ex-chip) with PAM4 BER ranging from $10^{-6}$ to $10^{-4}$. These lines cover a total spectral bandwidth of 1.2 THz, almost the same as that of the 100 GHz device with zero voltage on the saturable absorber (Fig. 4a). The device with 163 GHz interline separation demonstrates 4 low-noise lines having a power of about 3.6 mW per line (ex-chip), two of which reach a BER of about $10^{-7}$. For the shortest device with interline separation exceeding 200 GHz, 3 low-noise lines having a power of about 1 mW per line (ex-chip) can be identified. They have the BER ranging from $10^{-4}$ to $10^{-2}$ which is still below the SD-FEC20% limit of forward error correction and indicates the presence of mode locking.

We see that an increase in the interline separation can lead to a decrease of the number of low-noise lines. This is expected, as a high interline separation is achieved with decreasing laser length, leading to a decrease of the total gain, which depends exponentially on the laser length. Lasing is achieved when the gain in the laser medium compensates for the internal and mirror losses. In structures with a lack of gain, only modes in the center of the gain spectrum dominate. To compensate for the lack of gain in shorter devices, the current density should be increased, which eventually leads to the saturation of the QD ground state and the filling of the QD excited state, which starts to dominate in the lasing spectrum. Figure 8a shows the LI (optical power versus current) curves of two laser chips with the same structure and with different lengths, together with the power conversion efficiencies (PCE), right axis. The kink in the dependencies indicates the onset of the unwanted emission at the excited state, which is illustrated in Fig. 8b. For the 295 $\upmu$m device, with 138 GHz interline separation, the onset of the excited state is observed at 100 mA, providing enough optical power from the ground state at lower currents. For the 188 $\upmu$m device with 216 GHz interline separation, the excited state starts to dominate already above 20 mA, and pure ground state operation is associated with small optical power. For even shorter devices, lasing starts on the excited state right away.

Thus, to develop comb lasers with higher interline separation, one needs to increase the modal gain of the structure or increase the reflectivity of the laser mirrors. The other option is to use mode locking in the colliding regime^30,46^ with a doubled cavity length and saturable absorption in the middle. This approach, however, implies strict requirements on the cavity symmetry with respect to the absorber position.

## Conclusions

We have developed a mode-locked comb laser with 100 GHz interline separation, providing 11 to 23 modes depending on the voltage on the saturable absorber. The modes show low RIN due to the mode-locking effect, making data transmission of a 106 Gb/s PAM4 signal possible with BER below the KP4-FEC limit. We have found a relation between BER and RIN that underlies a Gaussian noise distribution. This allows us to evaluate the expected BER of the data transmission schemes involving the comb laser for other data formats, in particular for NRZ. We show that BER strongly depends on the power of the mode and reaches $10^{-10}$ for the modes with the highest intensity. We demonstrate the feasibility of practical DWDM data transmission schemes up to 2.3 Tb/s with a single comb source. The scheme employs signal amplification after the PIC using a single QD SOA. The scheme guarantees data transmission of a PAM4 signal with BER below modern FEC limits and error-free data transmission of an NRZ signal. We have also demonstrated the performance of comb lasers with higher interline spacing, in particular 138, 163, and 216 GHz, and their suitability for data transmission.

## Data Availability

The datasets used and analysed during the current study are available from the corresponding author on reasonable request.
